# In vitro evaluation of milk incorporated with *Eryngium billardieri* Delar alcoholic extract for antioxidant and antidiabetic potentials

**DOI:** 10.1002/fsn3.4339

**Published:** 2024-07-17

**Authors:** Mohadeseh Haghani, Vajiheh Fadaei, Mania Salehifar

**Affiliations:** ^1^ Department of Food Science and Technology, Shahr‐e‐Qods Branch Islamic Azad University Tehran Iran

**Keywords:** antioxidant activity, enzyme inhibition, *Eryngium billardieri*, functional milk

## Abstract

The present study evaluates the effect of *Eryngium billardieri* alcoholic extract (EBAE) at weight/volume levels of 0.5% and 1% on some physicochemical, sensory, and microbial properties of cow milk. Results showed that adding EBAE reduced the samples' pH, lightness index (L*), and whitening index (WI). Besides, it increased the levels of acidity, dry matter, viscosity, color indices a* and b*, total phenol content (TPC), total flavonoid content (TFC), antioxidant activity (AA), and enzyme inhibitory activity (EIA) of the fortified milk samples than the control sample (*p* < .05). During the storage period, a decrease in TPC, TFC, AA, and EIA was observed in all samples (*p* < .05). There was a positive direct relationship between the health activity of EBAE and its concentration. Moreover, EBAE was able to reduce the total microbial count (TMC) in the milk samples (*p* < .05). The sample containing 1% w/v of EBAE had the highest TPC, TFC, AA, and EIA but the lowest TMC, and it was highly acceptable in terms of sensory evaluation. Therefore, this sample was selected as the best treatment in this study. The results of this study confirm that EBAE may be used to improve antioxidant activity and beneficial effects in milk.

## Graphical abstract

*Eryngium billardieri* alcoholic extract (EBAE) can be used to improve the health properties of milk. *Eryngium billardieri* alcoholic extract (EBAE) provided milk with improved enzyme inhibitory activity. *Eryngium billardieri* alcoholic extract (EBAE) can increase the antioxidant activity of milk.

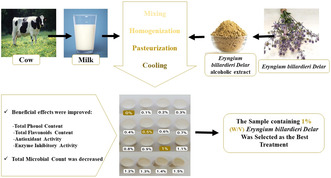

## 1 INTRODUCTION

Given the importance of medicinal plants in people's health, their use in producing functional foods has received much attention. Using these plants in treating diseases and in the pharmaceutical and food industries has long been of interest as several countries have taken measures to invest and plan their cultivation and mass production. Eryngium is a medicinal plant, a genus of flowering plants in the family Apiaceae. Apiaceae is one of the biggest plant families in the world, the members of which are known as vegetables and culinary plants with medicinal properties. Moreover, they contain various compounds with numerous biological activities. Apiaceae has three common species: *Eryngium billardieri*, *Eryngium bungei*, and *Eryngium caucasicum* (Paşayeva et al., 2017).

Eryngium contains tannin, sucrose, saponin, and alkaloid. It also contains other compounds, such as inulin, acetylene, flavonoids, coumarone, triterpenes, and monoterpene. The flavonoids present in Eryngium are quercetin and catechin. These compounds have antioxidant activity and prevent the destruction of cells through oxidative action. Therefore, they can repair damaged β‐cells and increase insulin levels from these cells. Other compounds identified in this plant are kaempferol, chlorogenic acid, and caffeic acid. The aerial parts of this plant contain lutein and β‐carotene, which are antioxidant and anti‐inflammatory compounds. The alcoholic extract of *E. billardieri*, which has alkaloid and antioxidant compounds, improved the liver function in rats with high cholesterol, although it had no side effects on their kidney function (Zarei et al., 2015). Fattahi and Hemayatkhah Jahromi (2016) confirmed the protective effects of the hydroalcoholic extract of Eryngium on hepatotoxicity caused by tricyclazole and attributed it to the presence of high phenolic compounds. The alkaloid compounds in this plant help to reduce fat absorbance from the intestine. Moreover, it has biological effects, such as anti‐inflammatory, antiapoptotic, antibacterial, antifungal, and antimalarial properties (Khani et al., 2021). Different parts of this plant are used to treat various diseases, including rheumatism, sinusitis, wound healing, urinary infections, scorpion stings, and goiter (Daneshzadeh et al., 2020). *Eryngium billardieri* is a plant whose root and aerial parts are recommended for treating diabetes. Golestaneh et al. (2021) reported that the extract of *E. caucasicum* improved the morphological damage of kidney tubular cells in diabetic rats. Afshari et al. (2019) reported that *E. caucasicum* extract had antidiabetic, hepatoprotective, and hypolipidemic effects in an animal model of type 2 diabetes (T2D). Khani et al. (2021) also reported that *E. billardieri* positively reduced lipid profile and liver enzymes, and controlled diabetes. According to Shirazi et al. (2020), the alcoholic extract of Eryngium can be effective in the treatment of diabetes, reduce malondialdehyde (MDA) concentration, and improve the antioxidant pathway of cells. The inhibitory effect of *E. billardieri* on some cancer cells has also been confirmed (Hasanbeiglu et al., 2022; Paşayeva et al., 2017; Roshanravan et al., 2018). To our knowledge, clinical studies have been carried out concerning Eryngium, especially on animals, but there has been no research on its use in functional food production.

Not all people like to consume herbal compounds or their extracts. Therefore, these compounds can be added to food to solve this problem. Adding *E. billardieri* alcoholic extract (EBAE) to cow milk can increase access to such a valuable substance. As a result, by providing the cultural backgrounds and introducing the functional products of this category, we can lower the consumption of unhealthy and non‐nutritious drinks, replace them with functional products, and improve public health. Hence, this study attempts to evaluate some physicochemical, sensory, and microbial properties and beneficial effects of cow milk containing EBAE.

## 2 MATERIALS AND METHODS

### 2.1 Materials

The aerial parts (i.e., stem, leaves, and flowers) of *E. billardieri* were prepared at the *Medicinal Plants Research Institute* of the Academic Center for Education, Culture, and Research of Karaj, Alborz, Iran, and raw milk was prepared at Pak Dairy Company (Tehran, Iran). All the chemicals needed for the tests were purchased from Merck Co. (Germany).

### 2.2 Preparation of EBAE

As mentioned earlier, the aerial parts (stem, leaves, and flowers) of Eryngium with the scientific name *E. billardieri* were obtained from the Medicinal Plants Research Institute of the Academic Center for Education, Culture, and Research of Karaj, Alborz, Iran. Also, they were approved by the Medicinal Plants Department of that research institute. The sample was mixed and dried in the shade under natural air and ambient temperature (about 24°C) for 10 days. Then, it was powdered using an electric mill. Extraction was performed using the percolation method and the percolator device. This process was performed using 50 g of powdered Eryngium and 250 mL of a solvent (70% ethanol). For the extraction process, first, the powder of Eryngium was mixed with 30% of the solvent (75 mL of 70% ethanol) at 24°C for 2 h, followed by transferring the mass uniformly into the percolator. The percolation process was performed for 72 h at 24°C. To evaporate the solvent from the extract, a rotary evaporator device was used under vacuum at a temperature of 55°C for 24 h (Golestaneh et al., 2021). In this respect, a sample of *E. billardieri* (No. TMRC‐2291) was stored in the Dry Herb Collection of the Traditional Medicine Research Center of Shahid Beheshti University of Medical Sciences (Tehran, Iran).

### 2.3 Production of milk samples containing EBAE

Cow milk (1.5% fat) was purchased from Pak Factory (Tehran, Iran). Then, an EBAE solution was prepared with two dilutions of 0.5% and 1% w/v. After mixing, the resulting mixture was homogenized at 40°C and 180 bar pressure. Finally, the produced samples were pasteurized at 75°C for 15 min and cooled to 10°C for 5 min. The prepared samples were kept at 4°C for 10 days (Abadi et al., 2023).

In this study, milk samples containing 0.5% and 1% w/v of EBAE based on primary production and sensory evaluation of the milk samples fortified with different weight–volume percentages of the extract (0.1, 0.20, 0.3, 0.4, 0.5, 0.6, …, 1.5) were selected. It is of note that the milk samples with EBAE percentages higher than 1% were not acceptable due to color and especially unpleasant taste. Besides, the initial production showed that the unpleasant taste of EBAE can be covered by adding natural sweeteners, such as licorice, berry juice, date juice, or stevia. In the case of using any of these sweeteners, higher amounts of EBAE can be added to the milk. Since the addition of sweetener was not part of the objectives of this study, the maximum weight–volume percentage of the added extract was considered 1.

### 2.4 Characterization of EBAE

In this research, pH was measured by a pH meter (Schott GERATE, Germany), and acidity was measured using the titration method (Abadi et al., 2023).

The aluminum chloride (AlCl_3_) colorimetric method was utilized to measure the total flavonoid content (TFC) at varying concentrations (100, 200, 300, 400, and 500 μg/mL). To this aim, the sample (1.5 mL) was mixed with AlCl_3_ solution (1.5 mL, 2% w/v), to which 5% potassium acetate (3 mL) was added. Following 40‐min incubation (Memmert, Germany), the absorption was determined at 415 nm (UV2100; USA), and TFC was expressed in milligrams of quercetin per gram (mg QE/g) of dry matter (Abadi et al., 2023).

Antioxidant activity (AA) was measured using a methanolic solution of stable 2,2‐diphenyl‐1‐picrylhydrazyl (DPPH) radical according to the method proposed by Abadi et al. (2023). To measure sample AA, a mixture of 100 μL of the sample (at 100, 200, 300, 400, and 500 μg/mL) and 3.9 mL of the 25 mM DPPH methanolic solution was prepared and kept in the darkness at room temperature. Following 30‐min incubation (Memmert, Germany), the absorption was determined at 517 nm (UV2100; USA) in comparison with that of the control solution. DPPH free radical inhibition percentage was calculated using Equation (1):

(1) $$\begin{array}{c} \text{Inhibition of DPPH}\;\left(\%\right)=\left(1–{\mathrm{Abs}}_{\text{sample}}/{\mathrm{Abs}}_{\text{control}}\right)*100 \end{array}$$

Abs_control_ = the absorbance of DPPH solution without extract; Abs_sample_ = the absorbance of DPPH solution with extract.

Finally, the results were expressed as IC_50_ (half‐maximal inhibitory concentration).

The sample total phenol content (TPC) at 100, 200, 300, 400, and 500 μg/mL was calculated using the Folin–Ciocalteu (FC) assay. To this end, in a test tube, the sample (0.25 mL) was mixed for 5 min with the FC reagent (2.5 mL) at room temperature. Freshly prepared sodium carbonate (Na_2_CO_3_) solution (1.5 mL, 2% w/v) was then added to the resulting mixture and maintained for 1 h. A spectrophotometer (UV2100; USA) was used to measure sample absorption at 760 nm. Besides, TPC was determined using a standard gallic acid curve (milligrams of gallic acid equivalent per gram (mg GAE/g) of dry extract) (Abadi et al., 2023).

The Bernfeld method was employed to determine the α‐amylase inhibitory activity (AIA). To prepare the reaction mixture, the sample (200 μL) was mixed with 0.02 M sodium phosphate (Na₃PO₄) (200 μL) with porcine pancreatic α‐amylase solution (PPA) (1.3 U/mL). It was then preincubated for 5 min at 37°C. Afterward, 4% starch solution (200 μL) was added to the buffer and incubated at 37°C for 10 min. This was followed by adding dinitrobenzoic acid solution (DNSA) (600 μL) to the resulting mixture, placing the mixture in a boiling water bath (for 7 min), and cooling it down in a cooling water bath. The mixture was then diluted with distilled water (1 mL), whose absorption was determined at 540 nm (UV2100; USA). The sample‐induced absorption was removed using an enzyme‐free (nonenzymatic) control solution (Abadi et al., 2023). The inhibition percentage was calculated from Equation (2):

(2) $$\mathrm{AIA}\;\left(\%\right)=\left(\left({\mathrm{Abs}}_{\text{control}}-{\mathrm{Abs}}_{\text{sample}}\right)/{\mathrm{Abs}}_{\text{control}}\right)*100$$

Abs_control_ = the absorbance of the control (100% enzyme activity); Abs_sample_ = the absorbance of the tested sample.

The α‐glucosidase inhibitory activity (GIA) was determined using Abadi et al. method (Abadi et al., 2023). To carry out the test, the sample (200 μL) was mixed with the rat intestinal α‐glucosidase crude enzyme solution (200 μL) at 0.2 U/m initial concentration in 67 mM phosphate buffer. Following 10‐min incubation (Memmert, Germany) at 37°C, 10 mM p‐nitrophenyl α‐D‐glucopyranoside (p‐NPG) solution (300 μL) was added to the obtained mixture, which was incubated at 37°C for 40 min. Then, sodium carbonate 100 (3 mL) was added to the resulting mixture so that the reaction can be stopped. The released p‐nitrophenol's absorption was determined at 405 nm (UV2100; USA). The inhibition percentage was calculated using Equation (3):

(3) $$\mathrm{GIA}\;\left(\%\right)=\left(\left({\mathrm{Abs}}_{\text{control}}-{\mathrm{Abs}}_{\text{sample}}\right)/{\mathrm{Abs}}_{\text{control}}\right)*100$$

Abs_control_ = the absorbance of the control (100% enzyme activity); Abs_sample_ = the absorbance of the tested sample.

The EBAE color was determined by spectrophotometer (Konica Minolta CM‐3500d), and the CIE Lab system was used to express color parameters L*, a*, and b* (Lima et al., 2021).

Whitening index (WI) was calculated using Equation (4) (Lima et al., 2021):

(4) $$\mathrm{WI}=100-\sqrt{{\left(100-L*\right)}^{2}+a{*}^{2}+b^{*2}}$$

### 2.5 Characterization of fortified milk samples

All tests were performed 1 and 10 days after producing fortified milk samples.

The pH of the samples was measured using a pH meter (Schott GERATE, Germany), and the titratable acidity (TA) of the milk samples was determined through titration (Abadi et al., 2023).

The viscosity of the samples was measured using the Brookfield Rotational Viscometer model DVII‐R (USA). Also, it was recorded at a speed of 60 rpm (revolutions per minute) and after 30 s of spindle rotation (UIA model LV) (Abadi et al., 2023).

Similar to EBAE, the color parameters of the milk samples were determined by a spectrophotometer (Konica Minolta CM‐3500d).

The milk samples' beneficial effects (i.e., TPC, TFC, AA, GIA, and AIA) were measured similarly to those of EBAE (Abadi et al., 2023); only at first, the milk sample was diluted with 50 mM potassium phosphate buffer in proportion to 5; then, 1 mL and 100 μL of diluted sample were used for the tests of enzyme inhibitory activity and other tests, respectively.

The total microbial count (TMC) was quantified according to the method proposed by Abadi et al. (2023) based on the pour‐plate method and using the plate count agar culture medium, and the number of colonies was reported in terms of log colony‐forming units per milliliter (log cfu/mL) of milk.

Sensory evaluation (flavor, color, consistency, smell, and overall acceptability) of the samples was performed by 50 untrained evaluators (20 men and 30 women in the age range of 20–65 years) using *a 5‐point Hedonic* scale scored as 5 (very good), *4* (*good*), *3* (*average*), 2 (*bad*), and 1 (*very bad*). It is noteworthy that all the participants liked consuming milk. Furthermore, they were aware that the milk samples were fortified with EBAE. They were not allergic to either milk or Eryngium.

### 2.6 Statistical method

A factorial experiment was used through randomized complete block design (RCBD) (for a sensory test) and completely randomized design (physicochemical tests and microbial test). The experiment had two factors, namely the alcoholic extract of eryngium factor (at three levels of 0%, 0.5%, and 1%) and the time factor (at two levels of the first and tenth days of storage). Three replications were considered for each treatment. In this study, the variance resulting from the difference between the panelists (in the sensory test) was removed by considering each panelist a block. In cases where there was a significant difference between the treatments, the least significant difference (LSD) test was used at the level of 0.05 to compare the means. After conducting the experiment based on the research and data collection methods, the data were analyzed using SAS 9.2.

## 3 RESULTS AND DISCUSSION

### 3.1 Physicochemical characteristics of milk samples containing EBAE during storage at 4°C

#### 3.1.1 pH and acidity

Adding EBAE and increasing its level caused a decrease in pH and an increase in acidity (Table 1) in the milk samples (*p* < .05). These outcomes can be attributed to the lower pH value and higher acidity of EBAE compared to cow's milk, i.e., 5.60% and 0.148%, respectively (Table 2); however, their values for milk were measured as 6.68% and 10.141%, respectively, in terms of lactic acid. The presence of acidic active compounds, such as chlorogenic acid and caffeic acid, can be the reason for the acidic pH of EBAE (Zarei et al., 2015).

**TABLE 1:** Physicochemical properties (titratable acidity, pH, and viscosity) of milk samples containing EBAE during cold storage (Mean ± standard deviation).

Characteristic	Treatment day	T1	T2	T3
Titratable acidity (lactic acid %)	1	0.139 ± 0.004^B,c^	0.174 ± 0.009^B,b^	0.213 ± 0.006^B,a^
	10	0.154 ± 0.003^A,b^	0.197 ± 0.007^A,b^	0.230 ± 0.009^A,a^
pH	1	6.70 ± 0.02^A,a^	6.54 ± 0.01^A,b^	6.49 ± 0.01^A,c^
	10	6.65 ± 0.02^B,a^	6.51 ± 0.01^B,a^	6.44 ± 0.02^B,b^
Viscosity (cP)	1	3.56 ± 0.02^A,c^	3.74 ± 0.02^A,b^	3.84 ± 0.03^A,a^
	10	3.66 ± 0.04^A,b^	3.83 ± 0.03^A,a^	4.08 ± 0.02^B,a^

*Note*: Different capital letters have significant differences in rows and different small letters have significant differences in columns (*p* < .05).

Abbreviation: EBAE, *Eryngium billardieri* alcoholic extract (%w/v), (T1: EBAE =0; T2: EBAE = 0.50; T3: EBAE = 1.00).

**TABLE 2:** Characterization of *Eryngium billardieri* alcoholic extract.

Characteristic	Amount
pH	5.60
Titratable acidity (lactic acid %)	0.148
TPC (mg GAE/g)	57.11
TFC (μg QE/g)	29.49
AA (mg/mL)	12.58
α‐Amylase inhibitory activity (%)	89.11
α‐Glucosidase inhibitory activity (%)	97.86
L*	13.33
a*	85.04
b*	93.78
Whitening index	−53.27

As time passed from the first day to the tenth day of storage in the refrigerator, the pH level of the milk samples decreased while their acidity level increased (*p* < .05). These results could be attributed to various reasons: (a) hydrolysis or spontaneous and slow degradation of proteins, (b) creation of favorable conditions for milk lactose fermentation and lactic acid production by microorganisms, (c) reactions between lactose and milk proteins and changes in the solubility of calcium phosphate, and (d) lipolysis and release of free fatty acids and changes in the solubility of divalent ions. In the milk sample containing polyphenols, pH decline is attributed to the degradation of polymeric phenols into monomeric phenolic acids with different acid strengths (pKa) through hydrolytic reactions and oxidative degradation (Wegrzyn et al., 2008).

Changes in the acidity and an increase in its level during shelf life in the milk samples fortified with additives have also been reported in other studies (Abadi et al., 2023; El‐Deeb, 2017; Veena et al., 2015; Wegrzyn et al., 2008).

#### 3.1.2 Viscosity

The increased level of EBAE in the milk samples increased their viscosity (Table 1) and this viscosity level also increased during the 10‐day storage period (*p* < .05). The increased viscosity of the milk samples could be due to the addition of EBAE and the impact of pH reduction on the agglomeration of milk proteins during storage (Veena et al., 2015) and the increased level of dry matter (unreported data). The increase in viscosity due to the increase in EBAE can be attributed to the reaction between milk compounds (such as proteins) and phytochemical compounds present in EBAE (e.g., polyphenolic compounds) (Sawale et al., 2015; Sharma et al., 2021).

Other researchers have also proven the increase in milk viscosity due to incorporating other plant extracts, such as *Pueraria tuberosa* and *Asparagus racemosus* (Sawale et al., 2015; Veena et al., 2015).

#### 3.1.3 Colorimetric analysis

Adding EBAE and increasing its level decreased L* (lightness index) and WI and increased a* (redness index) and b* (yellowness index) (Table 3) in the milk samples (*p* < .05). With the passage of time from the first day to the tenth day of storage of the samples, the redness and yellowness indices gradually increased while WI decreased (*p* < .05), and a slight decrease in lightness index was observed (*p* > .05).

**TABLE 3:** Physicochemical properties (color index) of milk samples containing EBAE during cold storage (Mean ± standard deviation).

Characteristic	Treatment day	T1	T2	T3
L*	1	83.15 ± 0.23^A,a^	77.28 ± 0.39^A,b^	70.89 ± 0.47^A,c^
	10	82.48 ± 0.53^A,a^	77.08 ± 0.19^A,b^	70.51 ± 0.50^A,c^
a*	1	2.32 ± 0.37^B,c^	3.19 ± 0.28^B,b^	6.03 ± 0.25^A,a^
	10	3.13 ± 0.19^A,c^	3.84 ± 0.17^A,b^	6.99 ± 0.14^A,a^
b*	1	3.02 ± 0.18^B,c^	6.64 ± 0.25^A,b^	17.08 ± 0.37^A,a^
	10	3.63 ± 0.27^A,c^	7.04 ± 0.31^A,b^	17.84 ± 0.46^A,a^
Whitening index	1	82.72 ± 0.26^A,a^	76.15 ± 0.09^A,b^	65.71 ± 0.53^A,c^
	10	81.83 ± 0.42^A,a^	75.76 ± 0.19^A,b^	64.93 ± 0.49^A,c^

*Note*: Different capital letters have significant differences in rows and different small letters have significant differences in columns (*p* < .05).

Abbreviation: EBAE, *Eryngium billardieri* alcoholic extract (%w/v) (T1: EBAE =0; T2: EBAE = 0.50; T3: EBAE = 1.00).

Research has shown that EBAE contains carotenoid pigments, especially β‐carotene (Khani et al., 2021). Because of these pigments, the values of L* (13.33) and WI (−53.27) were relatively low, and those of a* (85.04) and *b* (93.78) were high in this extract (Table 2). Therefore, adding this extract had a significant effect on the color parameters of the milk samples. The presence of phenols in EBAE may also be responsible for discoloration, especially reduced lightness in the milk samples (Sharma et al., 2021). Hydroxymethylfurfural (HMF) is a colored compound that can change the product's color over time. This compound is produced slowly and slightly in milk during the storage period due to the breakdown of sugars (Sharma et al., 2021). The presence of flavonoids increases the yellow color and creates red and orange colors in the product (Hussein & Safinaz, 2013). In this study, TFC in EBAE was calculated as 29.49 ± 0.95 mg QE/g (Table 1).

In line with the results of this study, changes in the color indices of the milk samples and their darkening due to the incorporation of plant extracts have been confirmed by other researchers (Abadi et al., 2023; Sawale et al., 2015; Veena et al., 2015; Wegrzyn et al., 2008).

### 3.2 Beneficial effects of milk samples containing EBAE during storage at 4°C

#### 3.2.1 Total phenol content (TPC), total flavonoid content (TFC), and antioxidant activity (AA)

The increased level of EBAE, as expected due to TPC and TFC in EBAE (Table 2), led to a significant increase in the TPC and TFC of the fortified milk samples (*p* < .05). In general, the addition of different levels of EBAE increased the TPC of the fortified milk samples (Table 4). This increase by 51.28%–61.92% was recoreded on the first day and 59.16%–67.82% on the tenth day compared to the control sample. The TFC also increased due to the addition of different levels of EBAE by 72.76%–83.18% on the first day and by 75.70%–86.46% on the tenth day (Table 4).

**TABLE 4:** Beneficial effects of milk samples containing EBAE during cold storage (Mean ± standard deviation).

Characteristic	Treatment day	T1	T2	T3
TPC* (mg GAE/mL)	1	42.17 ± 1.75^A,c^	86.56 ± 1.48^A,b^	110.74 ± 0.98^A,a^
	10	30.22 ± 1.32^B,c^	73.99 ± 1.07^B,b^	93.92 ± 1.78^B,a^
TFC** (μg QE/mL)	1	15.05 ± 0.24^A,c^	55.24 ± 0.89^A,b^	89.47 ± 0.75^A,a^
	10	10.63 ± 0.31^B,c^	43.74 ± 0.40^B,b^	78.49 ± 1.19^B,a^
AA*** (μL/mL, IC_50_)	1	221.26 ± 2.99^B,a^	83.65 ± 1.50^B,b^	59.57 ± 1.84^B,c^
	10	273.92 ± 3.09^A,a^	154.45 ± 3.45^A,b^	103.07 ± 2.34^A,c^
α‐Amylase inhibitory activity (%)	1	7.92 ± 0.40^A,c^	16.93 ± 0.13^A,b^	30.52 ± 0.58^A,a^
	10	6.98 ± 0.36^B,c^	13.79 ± 0.25^B,b^	27.85 ± 0.71^B,a^
α‐Glucosidase inhibitory activity (%)	1	14.32 ± 1.43^A,c^	38.79 ± 1.55^A,b^	67.74 ± 2.48^A,a^
	10	6.47 ± 1.28^B,c^	30.89 ± 1.71^B,b^	53.85 ± 2.09^B,a^

*Note*: Different capital letters have significant differences in rows and different small letters have significant differences in columns (*p* < .05).

Abbreviations: AA***, antioxidant activity; EBAE, *Eryngium billardieri* alcoholic extract (%w/v) (T1: EBAE =0; T2: EBAE = 0.50; T3: EBAE = 1.00); TFC**, total flavonoid content; TPC*, total phenol content.

The TPC and TFC of the milk samples decreased during their 10‐day storage period at refrigerator temperature (*p* < .05), which can be attributed to destructive oxidative reactions (Khan et al., 2017). The explanation put forth describes the presence of oxidative enzymes such as peroxidases and polyphenol oxidases in milk, which can cause the oxidation of phenolic and flavonoid compounds over time (Morales‐de la Peña et al., 2017). In addition, the reaction between phenolic compounds and milk proteins can justify the decreased content of these bioactive compounds in the samples during storage (Han et al., 2019; Yildirim‐Elikoglu & Erdem, 2018). The TPC level declined in the control sample, the sample containing 0.5% EBAE, and the sample containing 1% EBAE by 28.34%, 14.52%, and 15.19%, respectively, during the 10‐day storage period. Furthermore, during the 10‐day storage period, the reductions in TFC in control, the sample containing 0.5% EBAE, and the sample containing 1% EBAE were calculated as 29.37%, 20.82%, and 12.27%, respectively.

Adding EBAE and increasing its level in the samples caused a decrease in the IC50 level (Table 4) and, thus, an increase in the AA level. These results can be attributed to the increase in the content of bioactive compounds such as phenolic and flavonoid compounds (*p* < .05). The addition of different levels of EBAE led to a 62.19%–73.08% reduction on the first day in IC50 of the fortified samples compared to the control; meanwhile, this reduction was calculated as 43.61%–62.37% on the tenth day. During the 10‐day storage period, an increase in IC50 and a decrease in AA of the milk samples were observed (*p* < .05). Oxidative degradation and reduction of bioactive compounds (e.g., phenolic compounds and flavonoids) in the milk samples during storage can justify the AA reduction in the milk samples during storage (Ibrahim Khalifa & Ahmed Noseer, 2019). During the 10‐day storage period, IC50 increased by 19.22%, 45.84%, and 42.20% in the control, the sample containing 0.5% EBAE, and the sample containing 1% EBAE, respectively.

Taghizadeh et al. (2020) and Daneshzadeh et al. (2020) observed significant levels of flavonoids and phenolic compounds in the residues of Eryngium essence and Eryngium extract, respectively. The important phenolic and flavonoid compounds in Eryngium include quercetin, catechin, kaempferol, chlorogenic acid, and caffeic acid. In addition, lutein and β‐carotene, which have antioxidant activity, have been found in the aerial parts of this plant (Khodaie et al., 2019; Sepanlou et al., 2019). Savci (2021) confirmed the presence of various phenolic compounds, including trans‐coumaric acid, cinnamic acid, myristicin, 4‐dihydroxybenzoic acid, and catechol, in the alcoholic extract of Eryngium. Hajimehdipoor et al. (2016) also reported significant antioxidant activity in the alcoholic extract of Eryngium. In another study, Eryngium's antioxidant and anti‐inflammatory activity was attributed to the presence of tannins, terpenoids, and flavonoids (Zarei et al., 2015).

In agreement with the present study, the increased TPC level in the milk samples fortified with blackberry leaf extract (Sangsopha et al., 2019) and propolis extract (El‐Deeb, 2017) was also confirmed. Likewise, Khan et al. (2017) and Sawale et al. (2017) reported a decrease in TPC and TFC in raw and pasteurized cow and buffalo milks and flavored milk containing Arjuna extract, respectively, during the storage period.

Furthermore, in agreement with the results of the present research, an increase in AA was reported in the milk samples fortified with compounds containing antioxidant activity (Abadi et al., 2023; Gad & El‐Salam, 2010; Sangsopha et al., 2019; Sawale et al., 2015; Veena et al., 2014; Wegrzyn et al., 2008). Khan et al. (2017) observed decreased AA in cow and buffalo milk samples during sorage time.

#### 3.2.2 Enzyme inhibitory activity (EIA)

The use of inhibitors of α‐glucosidase and α‐amylase enzymes can delay the absorbance of carbohydrates through competitive inhibition. As a result, disaccharide hydrolysis and glucose absorbance are inhibited (Vichayanrat et al., 2002). The increased level of EBAE caused an increase in α‐glucosidase inhibitory activity (GIA) and α‐amylase inhibitory activity (AIA) (Table 4) in the milk samples (*p* < .05). Also, their EIA decreased during the 10‐day storage period at refrigerator temperature due to the decreased content of bioactive compounds (*p* < .05). In general, the addition of different levels of EBAE led to an increase in AIA by 53.22%–74.05% on the first day and by 49.38%–74.94% on the tenth day in the fortified samples compared to the control sample; in addition, GIA increased by 63.08%–78.86% on the first day and 79.05%–87.99% on the tenth day in the fortified samples compared to the control sample. During the 10‐day storage period, AIA decreased by 11.87%, 18.55%, and 8.75% in the control sample, the sample containing 0.5% EBAE, and the sample containing 1% EBAE, respectively; furthermore, GIA decreased by 54.82%, 20.37%, and 20.50% in the control sample, the sample containing 0.5% EBAE, and the sample containing 1% EBAE, respectively.

Previous studies have confirmed the significant antidiabetic activity of the extracts derived from the root and aerial parts of the Eryngium (Kheirollahzadeh et al., 2022; Küpeli et al., 2006). Khani et al. (2021) attributed the antidiabetic activity of Eryngium root extract to the various bioactive compounds (e.g., alkaloids, saponins, flavonoids, terpenoids, coumarone, chlorogenic acid, caffeic acid, and β‐carotene) present in this root. Khodaie et al. (2019) attributed this effect to quercetin and catechin (which are important flavonoids in the Eryngium). The blood sugar and blood fat‐lowering properties of some EBAE compounds, such as flavonoids and tannins, were reported in another study (Oyedemi et al., 2012). Hosseini et al. (2021) also attributed the antidiabetic activity of EBAE in rats to the presence of flavonoid compounds and possibly to the increase in insulin and serum malondialdehyde. Gulcin et al. (2019) reported that caffeic acid in cinnamon extract inhibits the activity of α‐glucosidase and α‐amylase.

#### 3.2.3 Correlation analysis of biological activity and phytochemical compounds evaluated in milk samples containing EBAE

The results of Pearson's correlation analysis are given in Table 5. The statistical analysis revealed a strong relationship between all the biological activities evaluated in this study and the TPC and TFC of the samples. All these relationships were significant at both 99% and 95% statistical levels. The relationship of TPC and TFC with EIA was positive and direct, while the relationship of TPC, TFC, and EIA with IC50 of the samples was negative and inverse. In general, an increase in the TPC and TFC of the samples led to a direct increase in GIA and AIA but a decrease in the IC50 level (which has an inverse relationship with AA). Here, the highest coefficient of determination (*r* ^2^) was related to the correlation between TFC and GIA (*r* ^2^ = .996).

**TABLE 5:** Pearson’ correlation coefficient between phytochemical compounds and biological activity.

Characteristic	TPC (mg GAE/mL)	TFC (μg QE/mL)	AA (μl/ml, IC_50_)	AIA (%)	GIA (%)
TPC (mg GAE/mL)	1				
TFC (μg QE/mL)	0.941**	1			
AA (μL/mL, IC_50_)	−0.982**	−0.890**	1		
AIA (%)	0.941**	0.996**	−0.888**	1	
GIA (%)	0.976**	0.988**	−0.937**	0.987**	1

*Note*: ** significant correlation at 1% confidence level.

Abbreviations: AA, antioxidant activity; AIA, α‐amylase inhibitory activity; GIA, α‐glucosidase inhibitory activity; TFC, total flavonoid content; TPC, total phenol content.

### 3.3 TMC results of milk samples containing EBAE during storage at 4°C

The increased level of EBAE in the milk samples increased the antimicrobial activity of the samples and decreased the TMC (Table 6) of the milk samples (*p* < .05). The TMC level of the milk samples increased significantly during the 10‐day storage period at refrigerator temperature. Nevertheless, there were fewer degrees of increase in TMC in the milk samples containing EBAE than in the control sample due to the antimicrobial effect of EBAE and the decrease in the growth and reproduction rate of microorganisms (*p* < .05). Overall, on the tenth day, TMC in the samples containing EBAE showed a decrease of about 1–2 logarithmic cycles compared to the control sample. The antimicrobial effect of EBAE can be attributed to its bioactive compounds. Eryngium contains phenolic acids (rosmarinic acid and chlorogenic acid), flavonoids (mainly kaempferol and quercetin glycosides), and saponins, which exhibit antibacterial and antifungal activities (Daneshzadeh et al., 2020).

**TABLE 6:** Total microbial count (TMC) of milk samples containing EBAE during cold storage (Mean ± standard deviation).

Characteristic	Treatment day	T1	T2	T3
TMC (Log CFU/mL)	1	3.23 ± 0.04^B,a^	3.07 ± 0.03^B,b^	3.04 ± 0.04^B,c^
	10	5.77 ± 0.06^A,a^	4.79 ± 0.09^A,b^	4.02 ± 0.08^A,c^

*Note*: Different capital letters have significant differences in rows and different small letters have significant differences in columns (*p* < .05).

Abbreviation: EBAE = *Eryngium billardieri* alcoholic extract (%w/v) (T1: EBAE =0; T2: EBAE = 0.50; T3: EBAE = 1.00).

Daneshzadeh et al. (2020) reported that the antioxidant and antimicrobial activities of the ethanol extract of the aerial parts of *E. billardieri* were positively related to its TPC and TFC. Allafchian et al. (2022) confirmed the antimicrobial activity of silver nanoparticles prepared with EBAE. In addition, Karimi et al. (2019) reported that EBAE exhibited antimicrobial and antifungal activities. Hajian‐Maleki and Shams‐Bakhsh (2023) also confirmed the antimicrobial effect of *E. billardieri* essential oil component.

### 3.4 Sensory evaluation of milk samples containing EBAE during storage at 4°C

The results revealed that adding EBAE did not significantly affect the milk samples' taste, consistency, and overall acceptability on the first day (Table 7). No significant difference in terms of smell was observed between the control sample and the sample containing 0.5% EBAE. However, adding 1% EBAE decreased the smell score in the milk samples. In terms of color, the score of the samples containing EBAE was lower than that of the control sample (*p* < .05). This difference is due to the milk's darker, yellower, and redder color caused by the EBAE incorporation, which can be attributed to the presence of carotenoid and flavonoid pigments in EBAE.

**TABLE 7:** Sensory characteristics of milk samples containing EBAE during cold storage (Mean ± Standard deviation).

Characteristic	Treatment day	T1	T2	T3
Flavor	1	4.83 ± 0.21^A,a^	4.83 ± 0.21^A,a^	4.50 ± 0.26^A,b^
	10	3.00 ± 0.00^B,b^	4.00 ± 0.00^B,a^	4.00 ± 0.00^A,b^
Color	1	5.00 ± 0.00^A,a^	4.14 ± 0.21^A,a^	4.00 ± 0.00^A,b^
	10	3.80 ± 0.26^B,b^	3.80 ± 0.26^A,a^	4.00 ± 0.00^A,b^
Texture	1	4.83 ± 0.21^A,a^	4.83 ± 0.21^A,a^	5.00 ± 0.00^A,b^
	10	4.00 ± 0.00^B,b^	4.67 ± 0.26^A,a^	4.67 ± 0.26^A,b^
Odor	1	5.00 ± 0.00^A,a^	4.83 ± 0.21^A,a^	4.50 ± 0.26^A,b^
	10	3.00 ± 0.00^B,a^	4.20 ± 0.16^B,a^	4.20 ± 0.16^B,b^
Overall acceptance	1	4.83 ± 0.21^A,a^	4.67 ± 0.26^A,a^	4.50 ± 0.26^A,b^
	10	3.00 ± 0.00^B,b^	4.00 ± 0.00^A,a^	4.00 ± 0.00^A,b^

*Note*: Different capital letters have significant differences in rows and different small letters have significant differences in columns (*p* < .05).

Abbreviation: EBAE, *Eryngium billardieri* alcoholic extract (%w/v) (T1: EBAE =0; T2: EBAE = 0.50; T3: EBAE = 1.00).

It should be noted that EBAE is cream‐brown in color. The score of sensory characteristics of the milk samples decreased during the 10‐day storage period, which may be attributed to proteolysis and production of volatile compounds, lipolysis and production of free fatty acids, oxidation of fats and production of aldehyde, alcohol and ketone compounds, and the activity of microorganisms and production of volatile nitrogen compounds. It is of note that the intensity of changes in sensory characteristics in the fortified milk samples was lower than that of the sample without EBAE (control) due to the significant antioxidant and antimicrobial activities of EBAE. Therefore, the best stability of sensory characteristics during the storage period was observed in the sample containing the highest level of EBAE (1%). According to the results of other researchers, milk fortification with nutritious ingredients affects the sensory properties of the final product. Therefore, the nutritious component should be added to the extent that it has the minimum negative impact on the sensory characteristics of the product (Abadi et al., 2023; El‐Deeb, 2017; Ibrahim Khalifa & Ahmed Noseer, 2019; Jalilzadeh‐Afshari & Fadaei, 2021; Sawale et al., 2015).

## 4 CONCLUSIONS

This study showed that EBAE has high TPC, TFC, AA, antimicrobial activity, and EIA. Therefore, the antioxidant, antimicrobial, α‐amylase, and α‐glucosidase enzyme inhibitory effects of this extract on the milk fortified with it during storage at 4°C were evaluated. The results showed that adding EBAE improved AA and EIA and decreased TMC during cold storage. In this respect, the shelf life of the control milk was estimated to be about 12 days, but it was 14 and 18 days, respectively, following an increase in the percentage of incorporated extract. Therefore, EBAE can be considered a source of natural antioxidants and inhibitors of α‐amylase and α‐glucosidase enzymes. Hence, it may be used to produce functional foods.

## AUTHOR CONTRIBUTIONS

**Mohadeseh Haghani:** Formal analysis (equal); investigation (equal); software (equal); visualization (equal); writing – original draft (equal). **Vajiheh Fadaei:** Conceptualization (equal); data curation (equal); investigation (equal); methodology (equal); project administration (equal); resources (equal); supervision (equal); validation (equal); writing – original draft (equal); writing – review and editing (equal). **Mania Salehifar:** Methodology (equal); supervision (equal); validation (equal); writing – review and editing (equal).

## CONFLICT OF INTEREST STATEMENT

The Authors declare that there is no conflict of interest.

## Data Availability

The data are available in the manuscript.
